# Branched-Chain Amino Acid Metabolic Reprogramming and Cancer: Molecular Mechanisms, Immune Regulation, and Precision Targeting

**DOI:** 10.32604/or.2025.071152

**Published:** 2025-12-30

**Authors:** Dongchi Cai, Jialin Ji, Chunhui Yang, Hong Cai

**Affiliations:** 1 Department of Clinical Laboratory, The Second Hospital of Dalian Medical University, Dalian, 116023, China; 2 Department of Clinical Laboratory, Anshan Central Hospital, Anshan, 114000, China; 3 Department of Pathology, The First Affiliated Hospital of Dalian Medical University, Dalian, 116021, China

**Keywords:** Branched-chain amino acids, metabolic reprogramming, tumor microenvironment, targeted therapy

## Abstract

Metabolic reprogramming involving branched-chain amino acids (BCAAs)—leucine, isoleucine, and valine—is increasingly recognized as pivotal in cancer progression, metastasis, and immune modulation. This review comprehensively explores how cancer cells rewire BCAA metabolism to enhance proliferation, survival, and therapy resistance. Tumors manipulate BCAA uptake and catabolism via high expression of transporters like L-type amino acid transporter 1 (LAT1) and enzymes including branched chain amino acid transaminase 1(BCAT1), branched chain amino acid transaminase 2 (BCAT2), branched-chain alpha-keto acid dehydrogenase (BCKDH), and branched chain alpha-keto acid dehydrogenase kinase (BCKDK). These alterations sustain energy production, biosynthesis, redox homeostasis, and oncogenic signaling (especially mammalian target of rapamycin complex 1 [mTORC1]). Crucially, tumor-driven BCAA depletion also shapes an immunosuppressive microenvironment, impairing anti-tumor immunity by limiting essential nutrients for T cells and natural killer (NK) cells. Innovative therapeutic strategies targeting BCAA pathways—ranging from selective small-molecule inhibitors (e.g., LAT1 and BCAT1/2) to dietary modulation—have shown promising preclinical and early clinical efficacy, highlighting their potential to exploit metabolic vulnerabilities in cancer cells while bolstering immune responses. By integrating multi-omics data and precision targeting approaches, this review underscores the translational significance of BCAA metabolic reprogramming, positioning it as a novel frontier in cancer treatment.

## 1 Introduction

Metabolic reprogramming is a hallmark of cancer, enabling tumor cells to sustain proliferation and adapt to hostile environments [1]. Among metabolic pathways, amino acid metabolism has gained prominence as a driver of tumorigenesis and tumor immunity [2]. In particular, the branched-chain amino acids (BCAAs)—leucine, isoleucine, and valine—have emerged as critical nutrients and signaling molecules in cancer. BCAAs serve as building blocks for protein synthesis and donate nitrogen for biosynthesis (e.g., nucleotide production) [3]. Cancer cells often become “addicted” to BCAAs, rewiring their uptake and catabolism to fuel growth and survival [4]. Here, we examine how BCAA metabolic reprogramming contributes to tumor development, progression, and metastasis, using breast and lung cancers as illustrative examples, while highlighting general principles across malignancies.

Studies have uncovered diverse roles for BCAA metabolism in cancer biology. Oncogenic signaling can upregulate BCAA transporters and enzymes, leading to altered intracellular BCAA pools that activate growth pathways (notably mechanistic target of rapamycin complex 1 (mTORC1)) and influence epigenetic and redox states [5,6]. In some tumors, BCAA catabolism is enhanced to provide energy and substrates for anabolic processes, whereas in others, BCAA breakdown is suppressed to accumulate leucine for signaling and protein synthesis [7]. These metabolic phenotypes can profoundly affect tumor behavior and patient outcomes. For instance, elevated expression of BCAA-catabolic enzymes or regulators has been correlated with aggressive disease in multiple cancer types [8,9], while impairing BCAA utilization can hamper tumor growth. Importantly, BCAA metabolism at the tumor site also shapes the immune microenvironment. Cancer cells consuming large quantities of BCAAs may deprive infiltrating T cells of these essential nutrients, dampening anti-tumor immunity [10]. Meanwhile, BCAA-derived signals (such as leucine sensing via mTOR) can modulate immune cell function and fate. Emerging evidence indicates that targeting BCAA metabolic pathways could enhance immune responses or overcome therapy resistance, underscoring the translational significance of this metabolic axis [11,12].

In this review, we detail the molecular mechanisms of BCAA metabolic reprogramming in cancer cells and the implications for tumor growth and metastasis. We discuss how BCAA availability and catabolism influence the tumor immune milieu. We also highlight multi-omics and clinical studies linking BCAA metabolism to cancer phenotypes, focusing on breast and lung cancers as primary examples. Finally, we summarize recent therapeutic strategies aimed at BCAA metabolism—from small-molecule inhibitors to dietary interventions—and consider how precise targeting of this pathway may yield new cancer treatments for cancer. This provides a comprehensive, up-to-date synthesis of a rapidly evolving field.

## 2 BCAA Metabolism: Pathways and Reprogramming in Cancer Cells

### 2.1 BCAA Catabolic Pathway Overview

Under normal physiology, BCAA homeostasis is tightly regulated by controlled uptake and enzymatic catabolism. Leucine, isoleucine, and valine are essential amino acids obtained from the diet or protein turnover. They enter cells via amino acid transporters such as the L-type amino acid transporter 1 (LAT1, encoded by SLC7A5), which in most normal tissues is low but allows high-capacity BCAA import when expressed [13]. Once inside the cell, BCAAs are reversibly transaminated by branched-chain amino acid transferases (BCATs) to yield corresponding branched-chain α-ketoacids (BCKAs) [14]. BCATs use α-ketoglutarate as the amino acceptor, producing glutamate in the process. Humans have two BCAT isoforms with distinct localization: cytosolic BCAT1 and mitochondrial BCAT2 [15]. BCAT1 is normally enriched in neural and germinal tissues but is ectopically expressed in various cancers [5], whereas BCAT2 is broadly expressed in adult tissues and resides in the mitochondria of many cell types [16].

Following transamination, the BCKAs (α-ketoisocaproate from leucine, α-keto-β-methylvalerate from isoleucine, and α-ketoisovalerate from valine) are irreversibly oxidized in the mitochondrial matrix by the multi-enzyme branched-chain α-ketoacid dehydrogenase complex (BCKDH) [17]. BCKDH catalyzes the rate-limiting step of BCAA catabolism, yielding acyl-CoA derivatives that enter central metabolic pathways. Leucine’s catabolites, acetyl-CoA and acetoacetate, can fuel the tricarboxylic acid (TCA) cycle or be diverted to lipogenesis; isoleucine’s breakdown produces acetyl-CoA and propionyl-CoA (the latter converted to succinyl-CoA); valine yields succinyl-CoA [9]. Through these conversions, BCAAs provide both carbon and nitrogen for cellular energy production and biosynthesis [18]. The activity of BCKDH is tightly regulated by a dedicated kinase (BCKDK) and a phosphatase (PPM1K, also called PP2Cm) [19]. BCKDK phosphorylates and inactivates BCKDH, suppressing BCAA breakdown, while PPM1K (which localizes to mitochondria) dephosphorylates and reactivates BCKDH. This regulatory loop allows cells to fine-tune BCAA catabolic flux according to nutrient availability and demand.

**Figure 1:**
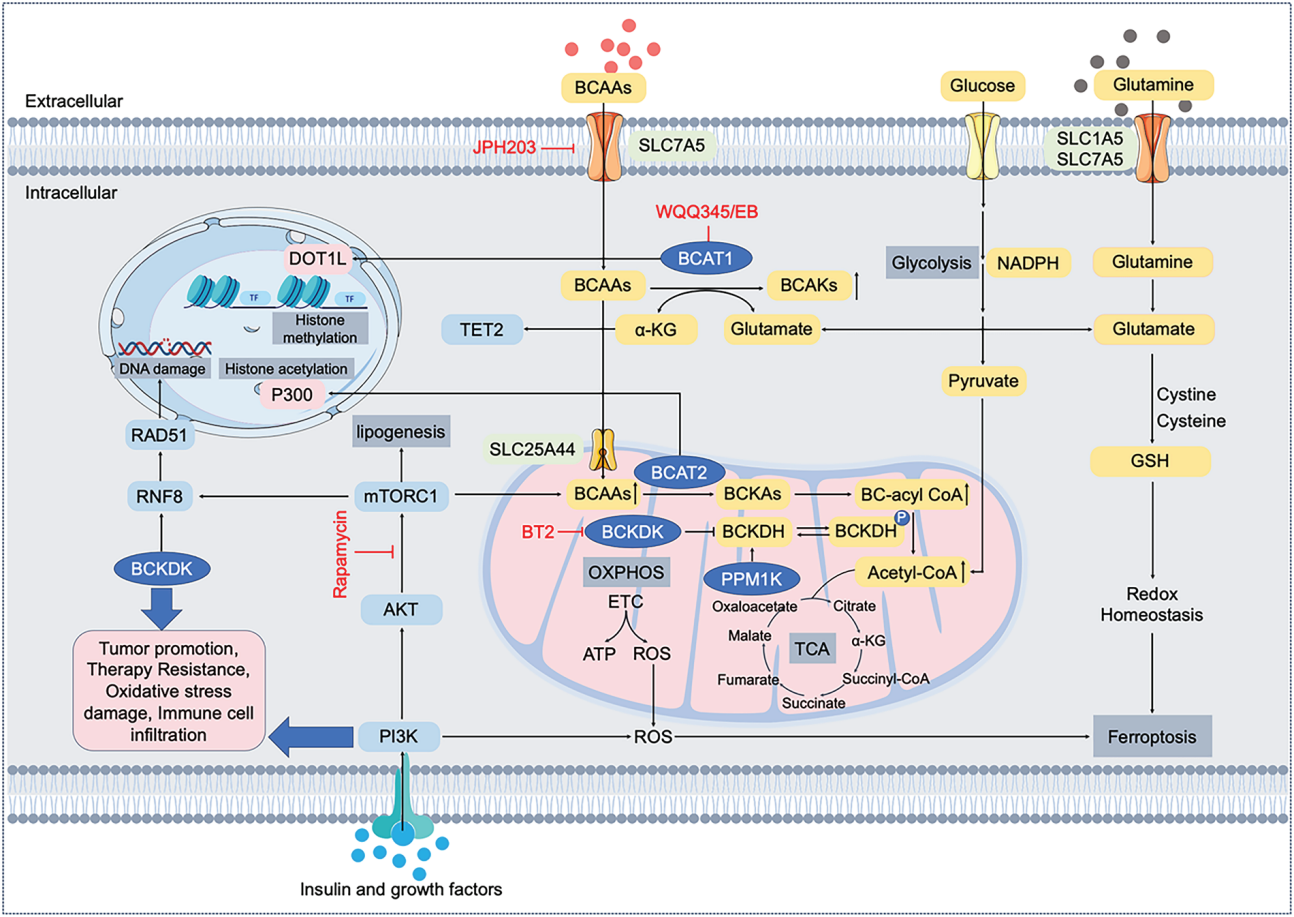
Molecular mechanisms and pathways of branched-chain amino acid (BCAA) metabolic reprogramming in cancer cells. BCAAs (leucine, isoleucine, valine) are transported into cancer cells mainly by LAT1 (encoded by SLC7A5). Intracellular BCAAs undergo transamination by branched-chain amino acid transaminases (BCAT1 cytosolic; BCAT2 mitochondrial), generating branched-chain α-keto acids (BCKAs) and glutamate. Subsequently, BCKAs are oxidized by branched-chain α-keto acid dehydrogenase (BCKDH), regulated by kinase BCKDK and phosphatase PPM1K, producing acetyl-CoA and intermediates for the tricarboxylic acid (TCA) cycle. BCAA metabolism intersects key signaling pathways such as PI3K/AKT/mTORC1, affecting histone modifications (DOT1L, P300), epigenetic regulation (TET2), DNA damage responses (RNF8/RAD51), lipogenesis (ACLY/FASN), oxidative phosphorylation (OXPHOS), reactive oxygen species (ROS) generation, and redox homeostasis (glutathione, GSH). Dysregulated BCAA metabolism thus contributes to tumor promotion, therapy resistance, oxidative stress tolerance, immune cell infiltration, and ferroptosis regulation. Therapeutic agents targeting LAT1 (JPH203), BCAT1 (WQQ345/EB), BCKDK (BT2), and mTORC1 (Rapamycin) are highlighted. LAT1, L-type amino acid transporter 1; BCAA, branched-chain amino acid; BCAT, branched-chain amino acid transaminase; BCKA, branched-chain α-keto acid; BCKDH; branched-chain α-keto acid dehydrogenase, BCKDK, branched-chain α-keto acid dehydrogenase kinase; PPM1K, protein phosphatase Mg^2+^/Mn^2+^-dependent 1K; TCA, tricarboxylic acid; PI3K, phosphoinositide 3-kinase; AKT, protein kinase B; mTORC1, mechanistic target of rapamycin complex 1; DOT1L, disruptor of telomeric silencing 1-like; TET2, ten-eleven translocation methylcytosine dioxygenase 2; RNF8, ring finger protein 8; OXPHOS, oxidative phosphorylation; ETC, electron transport chain, ROS, reactive oxygen species; GSH, glutathione; α-KG, α-ketoglutarate

In normal cells, these processes maintain BCAA levels within a narrow range and prevent toxic accumulation or depletion. For example, when BCAAs are abundant, BCKDK activity increases to curb excessive catabolism, whereas during starvation, PPM1K helps maximize BCAA oxidation for energy. In contrast, cancer cells often subvert these regulatory mechanisms [20]. Fig. 1 provides an overview of the BCAA catabolic pathway in normal cells and highlights the key points of oncogenic reprogramming (e.g., upregulation of LAT1, BCAT1, and BCKDK, and downregulation of PPM1K).

### 2.2 Oncogenic Reprogramming of BCAA Metabolism

Cancer cells hijack BCAA metabolism to satisfy their heightened anabolic needs. One common strategy is to increase BCAA uptake. Many tumors overexpress LAT1 at the cell membrane, facilitating high rates of leucine import [21,22]. Elevated LAT1 at the plasma membrane sustains intracellular leucine pools that directly engage the Rag–mTORC1 axis on lysosomal membranes, thereby promoting cap-dependent translation and cell-cycle progression [23]. In parallel, tumors frequently alter the expression of BCAT and BCKDH enzymes. At the subcellular level, BCAT1 (cytosol, upregulated in many cancers) accelerates cytosolic transamination to generate glutamate and BCKAs, while BCAT2 (mitochondria) supports coupling of transamination to mitochondrial oxidation. Notably, BCAT1—normally low in adult somatic tissues—is aberrantly overexpressed in many tumors, including breast, lung, prostate, and glioma [24]. High BCAT1 levels are associated with tumor proliferation and invasion, partly through activation of PI3K/Akt/mTOR and Wnt/β-catenin signaling cascades [25]. By increasing BCAT1, cancer cells rapidly transaminate imported BCAAs, impacting multiple downstream processes. The transamination yields glutamate (which feeds into glutathione synthesis for redox balance) and generates BCKAs that can be further oxidized for energy. BCAT1-driven catabolism has been linked to enhanced growth: for instance, chronic lymphocytic leukemia cells are “avid consumers” of BCAAs, using BCAT1 bidirectionally to both degrade BCAAs and regenerate them as needed [26]. Similarly, in glioblastoma, BCAT1 upregulation (via the histone methyltransferase DOT1L) increased BCAA catabolism and supplied glutamate for antioxidant defenses, promoting tumor cell proliferation under stress [27].

On the other hand, some cancers favor accumulating BCAAs rather than fully breaking them down. This is often achieved by suppressing BCKDH activity through upregulation of BCKDK or downregulation of PPM1K. By inhibiting the irreversible oxidation of BCKAs, tumor cells accumulate BCAAs and their ketoacid derivatives in the cellular pool. The surplus leucine can continuously engage mTORC1 signaling, supporting protein synthesis and growth [28]. Moreover, abundant BCAAs provide readily available building blocks for protein and nucleotide synthesis during rapid proliferation. Indeed, studies have found that high BCKDK and low PPM1K (indicating suppressed BCAA catabolism) are associated with more aggressive cancers and poorer patient survival [29]. In breast cancer, for example, invasive tumors showed significantly elevated BCKDK and reduced PPM1K compared to normal tissue, correlating with higher relapse rates [28]. This implies that restraining BCAA breakdown is advantageous for certain tumors, likely by preserving critical nutrients and signaling molecules.

BCAA metabolic reprogramming is heterogeneous across cancer types. Some tumors (e.g., pancreatic cancers or certain leukemias) exhibit increased BCAA catabolism to derive energy and macromolecular precursors, whereas others (e.g., subsets of breast cancers) show decreased BCAA catabolism leading to BCAA accumulation [29,30]. These differences reflect the metabolic demands and microenvironmental constraints each tumor faces. Nevertheless, a unifying theme is that BCAAs are indispensable for cancer cell survival and growth—whether as fuel, as signaling cues, or as substrates for biosynthesis [31]. Tumors dynamically adjust BCAA uptake and enzyme activity to maintain this balance. Altered BCAA metabolism, in turn, influences numerous oncogenic processes: it modulates signal transduction (mTORC1 and beyond), affects the epigenetic landscape (via changes in α-ketoglutarate availability), and helps maintain redox homeostasis by supplying glutamate for glutathione and nicotinamide adenine dinucleotide phosphate (NADPH) generation [32]. Through these mechanisms, reprogrammed BCAA metabolism directly contributes to tumor cell proliferation, survival under stress, and adaptation to nutrient-poor or hypoxic conditions.

In summary, cancer cells rewire the BCAA metabolic network at multiple points—from transporter upregulation (importing more leucine) to enzyme modulation (tuning BCAT and BCKDH activity)—in order to optimize BCAA utilization for malignant growth (Fig. 1). The following sections will illustrate how these alterations drive tumor progression and interact with other cancer hallmarks, with specific examples from breast and lung tumors.

## 3 BCAA Reprogramming in Tumor Progression and Metastasis

BCAA metabolic reprogramming is not just a bystander of tumor growth, but an active contributor to cancer progression and metastasis. Altered BCAA utilization can grant cancer cells with growth advantages, promote therapy resistance, and provide metabolic flexibility for invasion and colonization of distant organs [33]. Below, we discuss key mechanisms by which dysregulated BCAA metabolism propels oncogenic progression, with examples across cancer types.

### 3.1 Fueling Rapid Proliferation

Fast-growing tumors have enormous demands for both energy and biosynthetic materials. BCAA catabolism can supply both. Oxidation of BCKAs in the TCA cycle provides ATP and intermediates for anabolic pathways. One striking example is pancreatic ductal adenocarcinoma (PDAC), a notoriously aggressive cancer [34]. PDAC cells upregulate BCAA uptake (via multiple solute carrier transporters) and rely on BCAA catabolic enzymes for proliferation. In one study, PDAC cells had significantly higher expression of BCAT2 and the BCKDH subunit BCKDHA than normal pancreatic cells, and knockdown of these enzymes selectively impaired PDAC cell growth [29]. Notably, suppressing BCKDHA in PDAC did not reduce TCA cycle metabolites or respiration, but it markedly inhibited fatty acid synthesis, indicating that PDAC diverts BCAA carbon into lipogenesis [35]. Thus, PDAC uses BCAAs as an alternative carbon source to power lipid biosynthesis for membrane production—a critical need for rapidly dividing cells. This unique dependence suggests that disrupting BCAA catabolism could selectively starve PDAC tumors of building blocks for growth. More generally, many cancers funnel BCAA-derived carbons into the TCA cycle or anabolic routes to sustain proliferation when more common fuels (like glucose) are scarce. Breast cancer provides an illustrative case: triple-negative breast cancer (TNBC), which lacks estrogen receptor (ER), progesterone receptor (PR), and human epidermal growth factor receptor 2 (HER2), often shows increased reliance on amino acids, including BCAAs [36]. Metabolomic and transcriptomic analyses indicate that TNBC tumors have higher expression of BCAA-catabolic genes on average than hormone receptor-positive tumors [28]. In TNBC patient cohorts, high BCKDK (the kinase that inactivates BCKDH) is correlated with significantly worse relapse-free survival, whereas high BCKDH (the phosphatase activating BCKDH) correlates with better outcomes [37]. This suggests that TNBCs, which suppress BCAA catabolism via BCKDK upregulation, tend to be more aggressive and prone to recurrence. Consistently, breast tumors often exhibit increased BCAA uptake and elevated intracellular BCAA levels compared to normal breast tissue. Metabolomic studies have found higher concentrations of BCAAs in breast cancer tissue and patient plasma relative to non-cancer controls [38]. This is accompanied by overexpression of BCAA transporters and enzymes. LAT1, the high-affinity leucine transporter, is upregulated in a substantial subset of breast cancers and has been linked to disease outcomes. For example, high LAT1 expression (particularly ER+ tumors) was associated with shorter survival and therapy resistance in one study [39].

Lung cancer, particularly non-small cell lung cancer (NSCLC), is another malignancy where BCAA metabolic reprogramming plays a significant role. NSCLC tumors are metabolically active and must adapt to fluctuating oxygen and nutrient conditions in the lung microenvironment [40]. Similar to breast cancer, lung cancers commonly upregulate BCAA uptake and leverage BCAA metabolism to support tumor progression and therapy resistance. Rapidly proliferating lung tumor cells have an elevated demand for essential amino acids, including BCAAs [41]. One of the best-characterized metabolic changes in lung cancer is the LAT1 overexpression. High LAT1 levels have been documented in both lung adenocarcinomas and squamous cell carcinomas, correlating with more advanced disease and poorer prognosis. In stage I lung squamous cell carcinoma, patients with LAT1-high tumors had a five-year survival of ~52%, vs. ~88% for those with LAT1-low tumors [42]. LAT1 is minimal in normal lung tissue, making it a cancer-selective transporter. By overexpressing LAT1, lung cancer cells ensure they can import leucine (and other large neutral amino acids like phenylalanine) to sustain growth signaling [43]. A continuous leucine supply via LAT1 activates mTORC1, promoting protein synthesis and cell survival in the nutrient-scarce tumor regions. Some lung cancers also overexpress other amino acid transporters (such as ASCT2 for glutamine), but LAT1 is particularly critical for BCAA intake and has attracted attention as a therapeutic target [44].

Beyond carbon supply, the nitrogen from BCAAs is equally important to cancer cells. Transamination of BCAAs yields glutamate, which feeds the glutamine pool and can support nucleotide synthesis and other nitrogen-requiring processes [45]. Some cancer cells effectively use BCAA-derived nitrogen for *de novo* synthesis of nucleotides and nonessential amino acids, reducing their dependence on extracellular glutamine [46]. In essence, high BCAA turnover gives tumors a metabolic edge, buffering them against nutrient fluctuations. A pan-cancer analysis of The Cancer Genome Atlas (TCGA) data found that BCAT2 gene amplification or overexpression is present in numerous tumor types and is often associated with worse survival [47]. This underscores that many malignancies benefit from ramping up BCAA catabolism to fuel their growth. The cytosolic transaminase BCAT1 is another key player. Normally low in breast tissue, BCAT1 is frequently elevated in breast tumors, especially more advanced or basal-like cancers [48]. High BCAT1 in breast cancer patients has been linked to poor prognosis [49]. In experimental models, BCAT1 overexpression promotes breast cancer cell proliferation and invasiveness, partly by supplying intermediates to the TCA cycle and activating mTOR signaling to enhance mitochondrial biogenesis [50]. Conversely, BCAT1 knockdown can reduce breast cancer cell growth and migration *in vitro* [51]. The importance of BCAT1 as a therapeutic target is highlighted by the discovery of a small-molecule BCAT1 inhibitor, eupalinolide B (EB), which induced apoptosis in TNBC cells and showed anti-tumor effects in preclinical models [36]. Targeting BCAT1 thus appears promising for TNBC and other subtypes with high BCAT1 activity. Intriguingly, BCAT1 may also contribute to a cancer stem cell phenotype in breast cancer. For example, interferon gamma (IFNγ) released by T cells has been reported to increase cancer stemness in breast cancer via nuclear factor kappa-B (NF-κB)/LAT1 pathway, and co-inhibition of LAT1 and BCAT1 reduced this effect [52]. Although complex, this finding ties BCAA metabolism to therapy resistance (since cancer stem-like cells are often drug-resistant) and to immune-tumor interactions.

### 3.2 mTORC1 Activation and Protein Synthesis

As discussed earlier, leucine is a potent activator of mTORC1, a master regulator of cell growth. By accumulating intracellular leucine (through increased uptake or reduced breakdown), cancer cells maintain chronic mTORC1 signaling [53]. Constitutive mTORC1 activity drives unchecked protein synthesis and provides a growth and survival advantage. In estrogen receptor-positive (ER+) breast cancer, for instance, cells adapt to nutrient stress by overexpressing lethal giant larvae protein 2 (LLGL2), which promotes leucine import and mTORC1 activation [54]. This adaptation allows breast cancer cells to continue proliferating even when extracellular nutrients are low. While leucine-driven mTORC1 signaling boosts tumor growth, it can also contribute to therapy resistance (as discussed in Section 4). For example, high leucine levels and LAT1 upregulation have been linked to resistance to the anti-estrogen drug tamoxifen in ER+ breast cancer models [55,56]. Reducing leucine availability suppressed proliferation and restored drug sensitivity in these models. These findings link BCAA metabolic reprogramming directly to treatment outcomes. They exemplify how tumors exploit leucine-mTORC1 signaling to progress, but also suggest they could be vulnerable to nutrient limitation strategies.

### 3.3 Epigenetic and Transcriptional Reprogramming

BCAA metabolism can influence the epigenetic landscape of cancer cells, altering gene expression programs that drive tumor progression. A central player in this crosstalk is α-ketoglutarate (α-KG), the co-substrate for a family of DNA and histone demethylases [57]. By consuming α-KG during transamination, BCAT1 can modulate α-KG levels and thus affect the activity of α-KG–dependent dioxygenases. In acute myeloid leukemia (AML), for instance, high BCAT1 in leukemic stem cells depletes α-KG and induces a DNA hypermethylation profile akin to IDH-mutant leukemias [30]. This aberrant hypermethylation promotes stem cell self-renewal and blocks differentiation, contributing to leukemia aggressiveness. In solid tumors, a different mechanism has been observed: BCAT1-driven BCAA catabolism can increase α-KG production under certain conditions, affecting histone methylation status [58]. In osimertinib-resistant NSCLC, resistant cells upregulated BCAT1, leading to metabolic changes that increased α-KG availability and caused demethylation of histone H3K27 [11]. The loss of H3K27 methylation derepressed a host of glycolysis genes, thereby boosting glycolysis and fueling tumor progression [59]. Thus, BCAT1 acted as an epigenetic modulator, reprogramming gene expression to favor a more aggressive, drug-resistant phenotype. While the details differ—AML sees BCAT1 limiting α-KG (resulting in hypermethylation), whereas NSCLC sees BCAT1 increasing α-KG (resulting in hypomethylation)—both cases illustrate that dysregulated BCAA metabolism can lock cells into a malignant state via epigenetic mechanisms. The outcome is enhanced tumor progression, either by maintaining stemness or by activating stress-adaptive pathways like glycolysis.

### 3.4 Invasion and Metastatic Potential

Metastatic cancer cells often exhibit unique metabolic adaptations. BCAA reprogramming has been implicated in the metastatic cascade of several tumors. A common theme is that metastatic cells are metabolically plastic–engaging multiple nutrient pathways (glucose, glutamine, BCAAs, etc.) to meet the demands of invasion, survival in circulation, and colonization of distant organs [60]. For example, highly metastatic breast cancer cells display elevated activity in pathways spanning glucose, glutamine, and BCAA metabolism compared to non-metastatic cells. This multiplicity allows them to thrive in varying microenvironments. With regard to BCAAs specifically, one study found that suppressed BCAA catabolism correlates with greater metastatic propensity: in TNBC patients, high BCKDK (and low PPM1K) levels were associated with increased risk of relapse/metastasis [61]. This suggests that TNBC cells which preserve their BCAAs (rather than fully catabolizing them) may be better equipped to migrate and seed new tumors—possibly by channeling those amino acids into pro-migratory signaling or by using them to survive oxidative and detachment stresses during metastasis.

Conversely, some findings indicate that an excess of extracellular BCAAs can, somewhat paradoxically, inhibit metastasis via effects on the tumor microenvironment. Chi et al. reported that raising systemic BCAA levels in mice (either by a high-BCAA diet or genetically via PPM1K knockout) unexpectedly suppressed lung metastasis of breast cancer cells [28]. The high BCAA milieu reduced the cancer cells’ migratory and invasive capacity *in vitro* and was accompanied by lower N-cadherin expression in tumors (N-cadherin is a marker of the mesenchymal, invasive phenotype) [62]. While counterintuitive, this highlights the complex role of BCAAs: moderate BCAA availability promotes tumor cell growth, but forcing BCAA overload may tip the balance in favor of anti-tumor immune responses or other inhibitory effects (as explored in Section 5). Direct pro-metastatic roles of BCAA regulators have also been documented. BCKDK, which suppresses BCAA breakdown, has been identified as a pro-metastatic factor in colorectal cancer and hepatocellular carcinoma (HCC) [63]. Mechanistically, Src kinase-mediated phosphorylation and activation of BCKDK in HCC cells promoted cell migration and activated the ERK pathway, linking suppressed BCAA catabolism to pro-metastatic signaling [64]. These examples show that both increased and decreased BCAA catabolism can facilitate metastasis, depending on context—metastatic cells modulate BCAA metabolism in whichever way optimally suits their needs (whether for energy, structural components, or signaling molecules) during their dissemination and colonization.

## 4 Therapy Resistance: BCAA Metabolism as a Shield against Treatment

**Figure 2:**
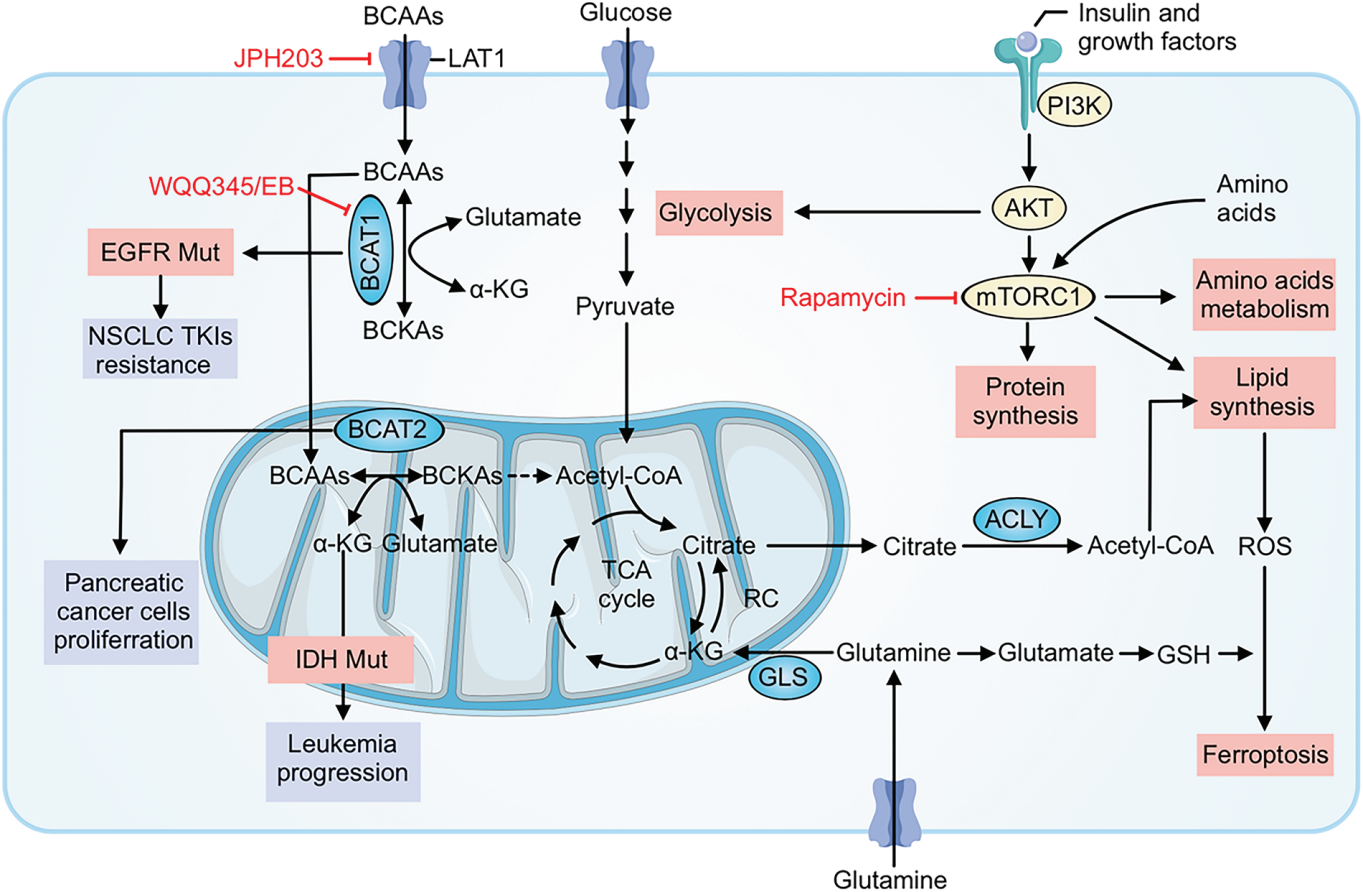
BCAA metabolic reprogramming fuels growth and multi-modal therapy resistance and highlights actionable nodes. Cancer cells increase leucine/isoleucine/valine uptake through LAT1 and channel BCAAs through BCAT1 (cytosol) and BCAT2 (mitochondria) to generate branched-chain α-ketoacids (BCKAs), acetyl-CoA and TCA-cycle intermediates. These fluxes sustain ATP production and mTORC1 signaling (activated downstream of PI3K–AKT and growth factors), which drives protein and lipid synthesis (via ACLY) and glycolysis, promoting survival under stress. Transamination also produces glutamate, replenishing α-ketoglutarate and glutathione (GSH) to buffer ROS, suppress ferroptosis, and blunt cytotoxic chemotherapy/radiation injury. In targeted-therapy settings, oncogenic programs (e.g., EGFR mutation) and acquired upregulation of BCAT1 maintain metabolic signaling and epigenetic states that support resistance to TKIs. Tumor-intrinsic amino-acid consumption further reshapes the microenvironment and can undermine antitumor immunity. Pharmacologic interventions indicated in red—JPH203 (LAT1 blockade), WQQ-345 or eupalinolide B (EB) (BCAT1 inhibition), and rapamycin (mTORC1 inhibition)—interrupt these circuits, decreasing anabolic supply lines, dampening mTORC1 output, restoring redox vulnerability, and thereby resensitizing tumors to chemo-, targeted-, and immunotherapies. BCAAs, Branched-chain amino acids (leucine, isoleucine, valine); LAT1, L-type amino acid transporter 1; BCAT1/BCAT2, Branched-chain amino acid transaminase 1/2; BCKAs, Branched-chain α-ketoacids; α-KG, Alpha-ketoglutarate; TCA cycle, Tricarboxylic acid cycle; ACLY, ATP-citrate lyase; GLS, Glutaminase; PI3K, Phosphoinositide 3-kinase; AKT, Protein kinase B; mTORC1, Mechanistic (mammalian) target of rapamycin complex 1; ROS, Reactive oxygen species; GSH, Glutathione; RC: Respiratory chain (electron transport chain); EGFR, Epidermal growth factor receptor; NSCLC, Non-small cell lung cancer; TKIs, Tyrosine kinase inhibitors; IDH, Isocitrate dehydrogenase; Acetyl-CoA, Acetyl coenzyme A; JPH203, Selective LAT1 inhibitor; WQQ-345/EB, BCAT1 inhibitors (WQQ-345; eupalinolide B); Rapamycin, mTORC1 inhibitor

Beyond driving tumor growth and spread, BCAA metabolic reprogramming contributes to resistance against various cancer therapies. Cancer cells rewire their metabolism to withstand the stresses imposed by chemotherapy, targeted therapies, and even immunotherapy [65,66]. Here, we detail how altered BCAA metabolism helps tumor cells evade the effects of treatment, and we illustrate these mechanisms in the contexts of cytotoxic therapy, targeted therapy, and immunotherapy (Fig. 2).

### 4.1 Resistance to Chemotherapy and Radiation

Conventional chemotherapy and radiation therapy kill cancer cells largely by inflicting DNA damage and generating reactive oxygen species (ROS), leading to oxidative stress and apoptosis [67]. Tumors with reprogrammed BCAA metabolism can blunt this oxidative stress, thereby surviving treatment. Enhanced leucine catabolism, in particular, yields metabolites that bolster cellular antioxidant defenses. Leucine’s transamination produces α-ketoisocaproate (α-KIC), which can directly scavenge ROS and increase the cell’s antioxidant capacity [68]. Furthermore, BCAA transamination via BCAT1/2 generates glutamate, a precursor for glutathione (GSH) synthesis. The resulting rise in intracellular glutamate drives cystine import and GSH production, which quenches chemotherapy-induced ROS and helps maintain redox homeostasis [69]. For instance, upregulation of BCAT2 in cancer cells elevates glutamate and GSH levels, thereby antagonizing ferroptosis (iron-dependent, ROS-mediated cell death) and protecting the cells from oxidative damage caused by treatment [70]. Additionally, BCAT1 itself contains a conserved Cys–X–X–Cys motif that can act as a redox switch to detoxify peroxides like H_2_O_2_, meaning BCAT1 overexpression directly buffers intracellular ROS [71].

Apart from boosting antioxidant molecule production, leucine-dependent signaling activates survival pathways. Persistent leucine/mTORC1 signaling promotes anabolic metabolism and upregulates anti-stress programs, including the expression of antioxidant enzymes and anti-apoptotic factors [72,73]. In essence, continuous mTORC1 activity helps tumor cells counteract the growth-inhibiting and pro-apoptotic signals triggered by chemo/radiotherapy. By sustaining ATP production, protein synthesis, and stress response gene expression, mTORC1 signaling enables cancer cells to weather the treatment-induced storm. Together, these metabolic adaptations raise the threshold for therapy-induced cell death. Tumors with high BCAA flux maintain redox balance despite ROS-generating treatments, making them less sensitive to cytotoxic agents. Supporting this, inhibiting BCAA-preserving mechanisms can resensitize tumors to therapy: for example, BCKDK inhibition in TNBC forced increased BCAA oxidation, depleted GSH, and stalled protein synthesis, which made the cancer cells more vulnerable to doxorubicin [37].

Another avenue through which BCAA regulators contribute to chemoresistance is by modulating DNA repair. Recent evidence suggests that BCKDK can translocate to the nucleus and enhance the repair of DNA double-strand breaks. In breast cancer cells, nuclear BCKDK was reported to promote homologous recombination repair (HRR) by stabilizing the RAD51 recombinase (via phosphorylation of the E3 ligase RNF8). This accelerated the repair of chemotherapy-induced DNA damage, aiding cell survival. Conversely, inhibiting BCKDK disrupted RAD51 stabilization and sensitized tumors to DNA-damaging agents. Although this is a non-metabolic function of BCKDK, it underlines how deeply embedded BCAA regulatory proteins are in the cellular networks that determine therapy response [45].

In summary, branched-chain amino acid reprogramming endows cancer cells with a multifaceted defense against chemo- and radiotherapy. By elevating antioxidant defenses (GSH and other ROS scavengers), maintaining energy and biosynthesis via mTORC1, and even enhancing DNA repair, tumor cells can survive levels of damage that would kill more metabolically naive cells. This explains why cancers with high BCAA metabolic activity often exhibit chemoresistance.

### 4.2 Resistance to Targeted Therapies (Endocrine and Kinase Inhibitors)

BCAA metabolism can also undermine targeted therapies, such as hormone-blocking treatments and kinase inhibitors. In ER+ breast cancer, we have seen that leucine-driven mTORC1 signaling can cause resistance to endocrine therapy (tamoxifen). Tamoxifen-resistant cells often upregulate LAT1 and increase leucine uptake, maintaining mTORC1 activity to bypass the need for estrogen signaling [55,56]. Targeting this metabolic adaptation—for instance, by limiting leucine or inhibiting LAT1—can restore sensitivity to hormonal therapy in preclinical models [54]. Thus, metabolic interventions may overcome certain forms of endocrine resistance.

Another clear example is in EGFR-mutant NSCLC treated with EGFR tyrosine kinase inhibitors (TKIs) like osimertinib. Tumors eventually develop resistance to TKIs, and metabolic reprogramming plays a role. Proteomic analyses of TKI-resistant NSCLC revealed consistent upregulation of BCAT1 in resistant cells [11]. Functionally, BCAT1 proved critical for maintaining the resistant state: its inhibition re-sensitized these cells to EGFR blockade. Mechanistically, BCAT1 reprogrammed cellular metabolism and epigenetics (increasing α-KG and promoting histone demethylation of glycolytic genes, as discussed in Section 3.3), thereby enabling cells to escape EGFR dependence. When BCAT1 was inhibited (for example, by the small-molecule WQQ-345), those metabolic and epigenetic changes were reversed, and previously resistant tumors responded to EGFR TKIs again [11,74].

These cases underscore a broader principle: when oncogenic signaling is inhibited by targeted drugs, cancer cells may turn to metabolic pathways like BCAA utilization to sustain survival. By cutting off that metabolic escape route—via BCAA metabolic inhibitors or dietary manipulation—we can potentially counteract resistance. Early-stage research is exploring combinations such as EGFR inhibitors with BCAT1 inhibitors in lung cancer, and anti-estrogen therapy with LAT1 inhibitors or leucine restriction in breast cancer.

### 4.3 Immune Evasion and Immunotherapy Resistance

Finally, BCAA metabolic reprogramming can impede the efficacy of immunotherapies by creating a nutrient-deprived, immunosuppressive tumor microenvironment. Cancer immunotherapy (like immune checkpoint blockade or CAR-T cell therapy) relies on functional, metabolically active T cells and NK cells to attack the tumor. However, tumors with high BCAA consumption effectively starve these immune cells. Tumor cells can deplete extracellular leucine, leaving infiltrating T cells without enough fuel to proliferate or activate their effector functions. Even if a checkpoint inhibitor unleashes the T cells, those T cells may fail to expand or produce cytotoxic molecules if they are leucine-starved. This metabolic form of immune evasion can manifest as primary or acquired resistance to immunotherapy.

There is evidence supporting this concept. A pan-cancer analysis found that tumors with high BCAT2 expression often had reduced T cell infiltration, consistent with a microenvironment less hospitable to T cells [47]. More directly, a recent preclinical study demonstrated that supplementing BCAAs can enhance T cell activity and improve responses to immunotherapy: in an animal model, adding BCAAs (to counteract tumor-imposed depletion) led to stronger CD8+ T cell effector function and synergized with anti-PD-1 therapy to better control tumor growth [31]. This suggests that, at least in some contexts, metabolic competition for BCAAs is a limiting factor for immunotherapy success.

On the other hand, simply feeding BCAAs to patients is a double-edged sword, as it might also nourish the tumor. Therefore, a more refined approach involves modifying immune cells to be less dependent on external BCAAs. A striking example is the engineering of CAR-T cells with a deleted BCKDK gene, which keeps their BCKDH complex active and allows them to utilize BCAAs more efficiently for energy [12]. These BCKDK-knockout CAR-T cells showed enhanced persistence and anti-tumor activity in solid tumor models, effectively overcoming the nutrient starvation imposed by the tumor. Such metabolic reprogramming of immune cells could make cell therapies much more potent in nutrient-poor tumor environments.

In summary, tumors can use BCAA metabolism as a shield not only against drugs and radiation but also against the immune system. By depleting key amino acids and activating stress resistance pathways, cancer cells make themselves less killable by both conventional therapies and immune-mediated attack. Recognizing and counteracting this phenomenon—through metabolic modulation of the tumor or the immune cells—represents a frontier in improving immunotherapy outcomes.

## 5 Immune Modulation via BCAA Metabolism in the Tumor Microenvironment

**Figure 3:**
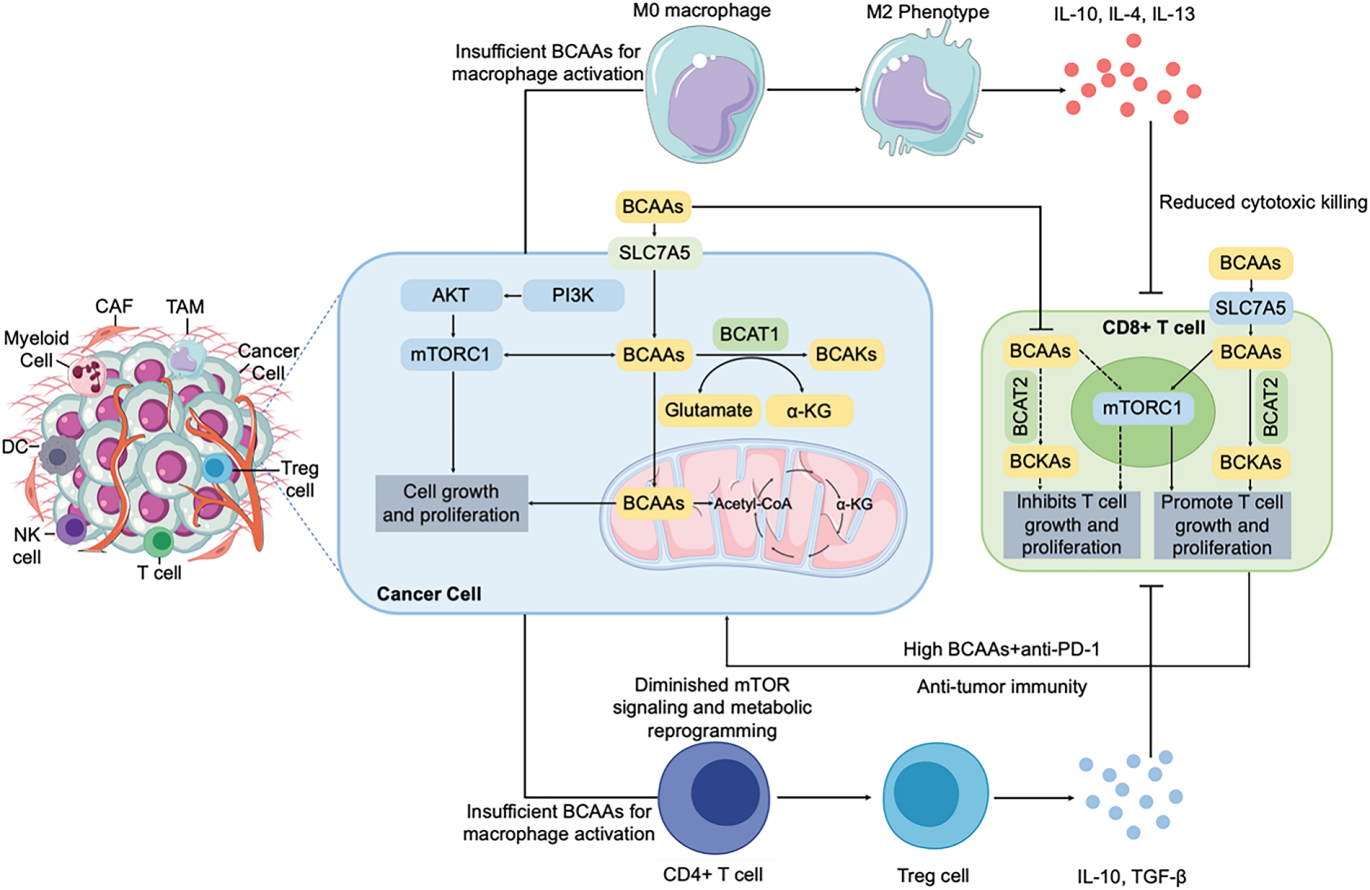
BCAA metabolism modulates the immune microenvironment in tumors. BCAA uptake and catabolism by cancer cells (via LAT1/SLC7A5 and BCAT enzymes) not only fuel tumor proliferation and survival through PI3K/AKT/mTORC1 signaling and mitochondrial metabolism but also profoundly shape the immune landscape of the tumor microenvironment. BCAAs availability regulates immune cell functions, especially T cells, through mTORC1 signaling. Low extracellular BCAA levels, driven by excessive uptake by cancer cells, suppress T-cell proliferation and effector functions, impairing anti-tumor immunity. Conversely, high extracellular BCAA concentrations, achieved by dietary supplementation or metabolic interventions combined with anti-PD-1 therapy, can promote T-cell proliferation and enhance anti-tumor immune responses. These metabolic interventions illustrate therapeutic potential in modulating the tumor immune milieu. BCAA, branched-chain amino acid; LAT1, L-type amino acid transporter 1; BCAT, branched-chain amino acid transaminase; BCKA, branched-chain α-keto acid; PI3K, phosphoinositide 3-kinase; AKT, protein kinase B; mTORC1, mechanistic target of rapamycin complex 1; PD-1, programmed cell death protein 1; CAF, cancer-associated fibroblast; TAM, tumor-associated macrophage; DC, dendritic cell; NK cell, natural killer cell; Treg, regulatory T cell; α-KG, Alpha-ketoglutarate; IL-10, interleukin-10; TGF-β, transforming growth factor-β

The dynamic interplay between tumor metabolism and the immune system is a frontier of cancer research, and BCAA metabolism sits at the heart of this crosstalk [75]. Immune cells, like cancer cells, require amino acids (including BCAAs) to function effectively. Thus, competition for BCAAs within the tumor microenvironment (TME) can profoundly shape immune responses. Additionally, the metabolic byproducts of BCAA catabolism and the signaling roles of BCAAs (e.g., via mTOR) directly influence immune cell differentiation and activity [76,77]. In this section, we focus on how tumor-driven changes in BCAA availability affect various immune cell types (Fig. 3).

### 5.1 T Cells: Nutrient Competition and Leucine Sensing

Solid tumors often create a nutrient-depleted TME by consuming available fuels. T cells infiltrating such tumors face scarcity of glucose, amino acids, and oxygen, which impairs their effector functions [78]. Rapidly proliferating tumor cells can deplete local leucine and other BCAAs, starving nearby T cells. Activated T cells, especially CD8+ cytotoxic T lymphocytes, have a high demand for leucine—they need leucine uptake to fuel clonal expansion and to activate mTORC1, which drives production of interferon-γ, granzymes, and other effector molecules [79,80]. If cancer cells with high LAT1 expression sequester most of the leucine, T cells in the vicinity become deprived of this essential nutrient. Consequently, their proliferation or cytokine production leads to a blunted immune attack on the tumor [81]. This scenario is analogous to tumors outcompeting T cells for glucose (the Warburg effect) or degrading tryptophan via IDO to impair T cell function. BCAA depletion in the TME thus diminishes T cell metabolic fitness and effector capacity, contributing to immune evasion by the tumor.

Leucine itself is also a critical signal for T cells. Upon activation, naive T cells dramatically upregulate amino acid transporters (including LAT1) to import leucine and other amino acids, which is required for turning on mTORC1 and shifting the T cells into a growth and effector mode [82]. In the absence of sufficient leucine, T cells fail to properly activate. For instance, genetic deletion of LAT1 in T cells leads to defective blast formation and severely reduced CD8+ T cell responses, due to the inability to uptake leucine and activate mTOR [83]. Different T cell subsets have distinct metabolic profiles: effector T cells rely heavily on anabolic metabolism and mTORC1 signaling, whereas regulatory T cells (Tregs) and memory T cells favor more oxidative metabolism and are somewhat less dependent on high exogenous leucine [84]. Nevertheless, BCAA metabolism appears important even for Tregs—BCAT1 is induced during human T cell activation and may influence the balance between effector and regulatory phenotypes [79]. In summary, leucine availability and mTORC1 signaling act as checkpoints for T cell activation and function. Tumors that induce leucine scarcity effectively push T cells into a nutrient-starved state, undermining anti-tumor immunity.

### 5.2 NK Cells: Innate Immune Surveillance and BCAAs

Natural killer (NK) cells, key innate immune effectors, are also influenced by BCAA levels. When activated (e.g., by cytokine IL-15), NK cells increase their metabolic activity similar to T cells—ramping up glycolysis and protein synthesis to support their cytotoxic functions [28]. In the TNBC study by Chi et al., boosting systemic BCAA levels (via diet or genetic means) significantly enhanced NK cell infiltration into tumors and their activity there. NK cells from high-BCAA conditions expressed higher levels of granzyme B and IFN-γ, indicating a stronger anti-tumor response. The rationale is that normally, tumor cells outcompete NK cells for nutrients in the TME. but in a BCAA-rich environment, NK cells can obtain sufficient leucine to sustain mTORC1 signaling and their effector program. Indeed, in Ppm1k-knockout mice (which have elevated circulating BCAAs due to defective BCAA catabolism), NK cells are better equipped to control tumor growth, evidenced by increased tumor cell apoptosis and slower tumor progression [28,85]. This suggests that metabolic interventions (like BCAA supplementation) might boost innate immune surveillance in some contexts. However, caution is warranted: excess leucine could simultaneously fuel tumor growth if cancer cells can efficiently utilize it. Therefore, any such strategy would need to be carefully timed or targeted, potentially providing short-term bursts of BCAAs to support immune cells during critical periods of an immune response.

### 5.3 Macrophages, MDSCs, and the Myeloid Compartment

Compared to lymphocytes, less is known about how BCAA availability impacts myeloid cells in the TME, such as tumor-associated macrophages (TAMs) and myeloid-derived suppressor cells (MDSCs). Macrophages can adapt their metabolism depending on their polarization: classically activated M1 macrophages (pro-inflammatory) rely mainly on glycolysis, whereas alternatively activated M2 macrophages (immunosuppressive) rely more on oxidative metabolism and mitochondrial respiration [86]. A tumor’s metabolic profile could influence this balance. For example, arginine depletion by tumor cells (via arginase) is known to push macrophages toward an immunosuppressive phenotype. By analogy, a low-BCAA environment might limit macrophage protein synthesis and cytokine production, impairing M1 polarization and tilting macrophages toward an M2-like, tumor-promoting state [87]. Conversely, an abundance of BCAAs might “re-energize” macrophages and dendritic cells, potentially enhancing antigen presentation and T cell stimulation. Direct evidence for BCAA effects on TAMs is sparse, but one can speculate that tumors with high BCAA catabolic activity create conditions that favor immunosuppressive myeloid cells. In support of this, a multi-omics study found that high BCAT2 expression in tumors correlated with reduced T cell infiltration and potentially higher levels of suppressive cells in some cancers [47,88]. While correlative, this finding is consistent with the idea that aggressive BCAA metabolism by tumors can help construct an immune microenvironment that is less hostile to the cancer. As for MDSCs, these cells thrive in and help maintain immunosuppressive environments. They are known to alter amino acid metabolism (for instance, MDSCs consume arginine and cystine and produce immunosuppressive metabolites). It is plausible that MDSCs are also influenced by BCAA levels or even contribute to BCAA depletion in the TME, although this hasn’t been explicitly shown yet. Future studies are needed to delineate how BCAAs affect the recruitment or function of MDSCs and TAMs.

In summary, tumor-driven BCAA metabolic changes reverberate through the immune system: starving T and NK cells of nutrients and potentially skewing macrophages toward tumor-friendly phenotypes. This creates a vicious cycle where an actively metabolizing tumor dampens immune attack, allowing it to grow unchecked.

## 6 Therapeutic Targeting of BCAA Metabolism in Cancer

The pivotal role of BCAA metabolism in tumor growth, metastasis, immune evasion, and therapy resistance makes it an attractive target for therapeutic intervention. Researchers are actively exploring a variety of strategies to disrupt or modulate BCAA availability and utilization in tumors. These include small-molecule inhibitors of BCAA transporters and enzymes, dietary interventions, and even metabolic engineering of immune cells to improve anti-tumor responses. The ultimate goal is to create therapies that selectively impair tumor metabolic fitness while sparing normal cells (or even enhancing immune cell function) [89,90]. Below, we group the major strategies and discuss their current status, applications, challenges, and future prospects (see also Table 1 for a summary).

**Table 1:** Recent therapeutic strategies targeting BCAA metabolism in cancer

Target/Strategy	Therapeutic agent or approach	Mechanism of action	Research type	References
Engineered T cells (Immunotherapy)	BCKDK-knockout CAR-T cells	Enhances CAR-T cell metabolic fitness by promoting BCAA catabolism, improving antitumor immunity	Preclinical	[12]
BCAT2 (Mitochondrial BCAA transaminase)	BCAT2 inhibitor	Blocks mitochondrial BCAA catabolism, limiting TCA cycle anaplerosis and cancer cell energy supply	Preclinical	[33]
LAT1 (Leucine Transporter)	JPH203 (KYT-0353), small-molecule inhibitor	Inhibits leucine uptake, suppressing mTORC1 signaling and tumor cell growth	Phase I/II	[91]
BCAT1 (Cytosolic BCAA transaminase)	Eupalinolide B (natural product inhibitor)	Inhibits BCAT1-mediated BCAA transamination, reducing α-KG production and impeding tumor metabolism	Preclinical	[11,91]
	WQQ-345 (synthetic inhibitor)			
BCKDK (BCKD kinase)	BT2, GSK180736A (allosteric BCKDK inhibitor) (genetic knockdown approaches)	Inhibits BCKDK, activating BCKDH to promote BCAA catabolism and disrupt metabolic reprogramming in cancer cells	Preclinical	[45,92]
Dietary BCAA Modulation	Low-BCAA or Low-Leucine Diet High-BCAA Supplementation	Alters systemic and tumor BCAA availability, affecting mTOR signaling, tumor metabolism, and immune responses	Preclinical/Exploratory	[93,94]

Note: BCAA, branched-chain amino acids; BCAT1, branched-chain amino acid transaminase 1; BCAT2, branched-chain amino acid transaminase 2; BCKDK, branched-chain α-keto acid dehydrogenase kinase; BCKDH, branched-chain α-keto acid dehydrogenase; CAR-T, chimeric antigen receptor T cells; LAT1, L-type amino acid transporter 1; mTORC1, mechanistic target of rapamycin 1; TCA (cycle), tricarboxylic acid cycle; α-KG, alpha-ketoglutarate.

### 6.1 Targeting BCAA Uptake: LAT1 Inhibitors

Because LAT1 is the principal gateway for leucine entry into many tumors, it has been a prime target for therapy. Blocking LAT1 essentially “cuts off the supply line” of leucine and other essential amino acids. The most advanced LAT1 inhibitor is JPH203 (also known as KYT-0353), a selective small-molecule inhibitor of LAT1 [95]. In preclinical studies, JPH203 potently suppressed proliferation of LAT1-high cancer cell lines and reduced tumor growth in animal models by inducing nutrient starvation stress. Importantly, LAT1 is expressed at high levels in tumors but only at minimal levels in most normal adult tissues (notable exceptions include the blood-brain barrier and some hepatocytes), suggesting a favorable therapeutic window [96]. JPH203 has progressed to human trials. A first-in-human Phase I trial in patients with advanced solid tumors (mainly biliary tract and pancreatic cancers) found that JPH203 was generally well tolerated; the dose-limiting toxicity was a reversible elevation of bilirubin, likely due to on-target effects of LAT1 inhibition in liver cells (LAT1 helps transport bilirubin conjugates) [97]. Notably, some patients achieved stable disease for several months on JPH203, and one patient with biliary tract cancer had a partial tumor response. These early signs of activity have led to a Phase II trial focusing on advanced biliary tract cancers, where LAT1 is often highly expressed [98].

Beyond JPH203, other LAT1 inhibitors are under development. For example, SKN103 is a novel LAT1-specific inhibitor reported to have strong anti-tumor effects in preclinical models [99]. Interestingly, several approved drugs or endogenous hormones can interact with LAT1 as transported substrates (e.g., levodopa and thyroid hormones) and therefore may show weak competitive effects *in vitro*, but they are not potent or selective enough to serve as clinical LAT1 inhibitors [100,101]. The main challenge for LAT1 inhibitors will be ensuring that blocking leucine uptake in tumors does not cause systemic malnutrition or prompt cancer cells to compensate by upregulating other transporters or scavenging nutrients from their environment. So far, the clinical data are encouraging that a balance can be struck: with careful monitoring of patients’ nutritional status and perhaps dietary support, LAT1 inhibition can be delivered safely. If effective, LAT1 inhibitors could be combined with other therapies (e.g., mTOR inhibitors or chemotherapy) for a one-two punch—cutting off nutrient supply and directly hitting growth signaling.

### 6.2 Targeting BCAT1 (Cytosolic Transaminase)

BCAT1 has emerged as a key metabolic enzyme in numerous tumors, particularly those that thrive in hypoxic or nutrient-stressed niches. Inhibiting BCAT1 is an attractive strategy because it can disrupt multiple pro-tumor processes at once: it slows BCAA catabolism (potentially starving the TCA cycle), it may cause toxic buildup of BCKAs, it reduces glutamate production (impacting redox balance and nucleotide synthesis), and it can restore normal α-KG levels (thereby potentially reversing certain epigenetic changes). Several BCAT1 inhibitors have been identified:

#### 6.2.1 ERG24

This compound is an analog of the naturally occurring amino acid ergothioneine and was one of the first BCAT1 inhibitors described [97]. ERG240 acts as a substrate mimic that binds to BCAT1’s active site, effectively clogging the enzyme. In IDH-wildtype AML models, ERG240 increased α-KG levels and induced cellular differentiation, mirroring the effect seen in IDH-mutant AML, where α-KG levels are high [30,102]. However, ERG240 had poor bioavailability and pharmacokinetics, limiting its practical use. Nonetheless, it served as a proof of concept that BCAT1 can be targeted and set the stage for designing better inhibitors.

#### 6.2.2 Eupalinolide B (EB)

Reported in 2021, EB is a sesquiterpene lactone isolated from a medicinal plant, identified as a direct BCAT1 inhibitor [91]. EB binds to BCAT1 and suppresses its activity, which in turn led to apoptosis of TNBC cells that depend on BCAT1-driven metabolism [36]. In mice bearing TNBC xenografts, EB treatment significantly reduced tumor growth. While these results are promising, EB itself is a relatively large and complex molecule (and being a natural product, it may hit multiple targets). Its drug-like properties are not ideal. Therefore, researchers are working on synthesizing simpler analogs of EB or entirely new compounds that capture its BCAT1-inhibitory effect with improved specificity and bioavailability [103].

#### 6.2.3 WQQ-345

This is a novel small-molecule BCAT1 inhibitor discovered through structure-based design and high-throughput screening [11]. WQQ-345 is cell-permeable and has favorable stability. It inhibits BCAT1 at low micromolar concentrations. What makes WQQ-345 especially interesting is its efficacy in a very challenging setting: EGFR TKI-resistant NSCLC (as discussed in Section 4.2). In mice with TKI-resistant lung tumors, WQQ-345 not only slowed tumor growth but actually caused regressions in combination with EGFR blockade—effectively re-sensitizing the tumors to the treatment they had previously evaded [11,74]. WQQ-345 reversed the BCAT1-driven metabolic and epigenetic changes that underpinned drug resistance. This compound is a strong candidate for further development, possibly as an adjunct to targeted therapies in resistant cancers. Collectively, these BCAT1 inhibitors demonstrate that targeting a single metabolic enzyme can have broad anti-tumor effects and even enhance other treatments. Moving forward, one could envision using a BCAT1 inhibitor to “soften up” a tumor before hitting it with chemotherapy or a targeted agent—the metabolic stress might make cancer cells less able to cope with the subsequent assault. Another potential application is in preventing metastasis or disease relapse. Since BCAT1 is implicated in supporting cancer stem cells and survival under stress, an inhibitor might keep disseminated tumor cells in check or make adjuvant therapies more effective. A key consideration for clinical trials will be selecting patients whose tumors are BCAT1-driven (e.g., by measuring BCAT1 expression or activity in the tumor) so that those most likely to benefit receive the treatment [104].

### 6.3 Targeting BCAT2 (Mitochondrial Transaminase)

Compared to BCAT1, the mitochondrial BCAT2 has received less attention, but it is crucial for BCAA catabolism in certain contexts (such as in PDAC, some liver cancers, and potentially in systemic BCAA metabolism related to cachexia). Researchers at GlaxoSmithKline recently developed potent and selective BCAT2 inhibitors based on a 2-aryl benzimidazole scaffold [105]. Through medicinal chemistry optimization, they achieved nanomolar-range inhibition of BCAT2. These compounds were initially tested in non-cancer settings: for example, in models of heart failure and cancer cachexia, where excessive BCAA breakdown is detrimental (heart failure patients often have hyperactive BCAA catabolism leading to cardiac dysfunction). In those models, BCAT2 inhibitors helped by raising circulating BCAA levels and reducing toxic BCKA accumulation, confirming on-target activity [106]. Translating BCAT2 inhibition to cancer means using it to push cancer cells into a metabolic corner—forcing them to either accumulate BCKAs or activate BCKDH and potentially overproduce ROS or deplete NAD+ (since BCKDH consumes NAD+). A recent preclinical study in 2023 did exactly this: they tested a BCAT2 inhibitor in a PDAC mouse model, since PDAC cells often have high BCAT2 expression. The BCAT2 inhibitor significantly slowed tumor growth and, intriguingly, induced markers of ferroptotic cell death in the tumors [69]. This aligns with separate findings that loss of BCAT2 can remove a cell’s protection against ferroptosis, making them more susceptible to iron-dependent oxidative cell death. Thus, BCAT2 inhibitors could potentially synergize with treatments that induce oxidative stress or ferroptosis in cancer cells. No BCAT2 inhibitor has reached clinical trials yet, and one reason is the safety concern: systemic BCAT2 inhibition would raise circulating BCAA levels (as seen in genetic BCAT2 or BCKDH deficiencies), which can lead to metabolic complications such as insulin resistance or neurologic issues akin to maple syrup urine disease [107]. Therefore, any cancer therapy targeting BCAT2 might require short-term dosing or targeted delivery. For example, encapsulating a BCAT2 inhibitor in a nanoparticle to concentrate its effect in the tumor could minimize systemic exposure and toxicity [108].

### 6.4 Targeting BCKDK (Activating BCKDH)

Inhibiting BCKDK is conceptually the flip side of what many tumors do. Tumors upregulate BCKDK to save their BCAAs; inhibiting BCKDK forces them to burn those precious amino acids. The prototype BCKDK inhibitor is a compound called BT2, which allosterically inactivates BCKDK. BT2 has actually been used in mice to treat diet-induced diabetes, as chronic BCKDK inhibition lowers BCAA levels and improves insulin sensitivity (high BCAAs are linked to insulin resistance) [92]. In cancer models, BCKDK inhibition has produced some encouraging results. As mentioned, in TNBC models, blocking BCKDK (with BT2 or by shRNA) led to slower tumor growth and enhanced the efficacy of chemotherapy [28,37]. Essentially, by preventing the tumor from hoarding BCAAs, BCKDK inhibition causes the tumor to over-oxidize BCAAs, which can create an energetic shortfall or metabolic byproducts that stress the cell. It may also deprive the tumor of leucine signaling for mTOR, compounding the anti-tumor effect [109]. Preclinical studies in HCC have also shown that turning BCKDH “back on” (via BCKDK inhibition or PPM1K activation) can suppress cancer aggressiveness. Tumors with active BCKDH cannot accumulate BCAAs and seem less able to invade or metastasize [110]. These findings make a compelling case for BCKDK as a target.

The challenge, as with BCAT2, is the shadow of maple syrup urine disease—a condition where BCKDH is overly active or BCKDK is deficient, leading to continuous BCAA breakdown and dangerously low BCAA levels in the body. Patients with MSUD suffer from neurological damage if not managed with a strict diet. A systemic BCKDK inhibitor could potentially induce an MSUD-like state if overdone [111]. However, in the context of cancer therapy, one might use a BCKDK inhibitor in short bursts (e.g., a few days at a time) or in a localized manner. Another approach is to tie the BCKDK inhibitor to a tumor-targeting moiety (like an antibody or a ligand that the tumor uptakes), thereby concentrating it in cancer cells. Additionally, since some tumors might have specific kinases that regulate BCKDK, blocking those kinases (for instance, blocking Src in an HCC where Src activates BCKDK [64]) could be a more tumor-selective way to achieve the same end. Interestingly, metabolic side effects of BCKDK inhibition might be mitigated by dietary support—if patients on a BCKDK inhibitor up their intake of BCAAs slightly (or overall protein), it might offset losses in normal tissues while still hurting the tumor. These are the kinds of nuances that will need to be ironed out in translational research [112].

### 6.5 Dietary and Nutritional Interventions

Because BCAAs come from the diet, adjusting what patients eat is a straightforward-sounding, yet powerful, tool. Researchers have explored both BCAA restriction and supplementation in different scenarios.

#### 6.5.1 BCAA Restriction

Limiting intake of BCAAs, especially leucine, can slow tumor growth in certain models. This dietary strategy essentially mirrors what drugs like LAT1 inhibitors do—deprive the tumor of leucine. In ER+ breast cancer models, a low-leucine diet not only slowed tumor growth but also made tumors more susceptible to tamoxifen treatment, delaying or preventing the onset of resistance [113]. In another study, BRAFV600E-mutant thyroid cancers exhibit distinct metabolic reprogramming characterized by enhanced one-carbon metabolism and upregulation of the BCAA transporter SLC7A5 [114]. However, one size does not fit all: Chi et al. found that raising dietary BCAAs could reduce metastasis in a breast cancer model (via immune effects) [28]. And in clinical contexts like cirrhotic patients at risk of HCC, BCAA supplementation (not restriction) improved outcomes, likely by improving nutritional status and perhaps immune surveillance [93]. These seemingly conflicting findings highlight that diet must be tailored. For tumors that clearly crave BCAAs (like many PDACs or some breast cancers), a medically supervised low-BCAA diet could be beneficial. This might involve special formulations of food or amino acid mixtures that omit BCAAs. But implementing such diets in patients is challenging—BCAAs are in many protein-rich foods, and malnutrition could result if not done carefully. There’s ongoing research into intermittent protein restriction (e.g., cyclic fasting or low-protein periods during chemo) as a means to weaken tumors at specific times while maintaining harm to patients [94]. Early trials of protein-restricted diets during therapy have shown they can be tolerated and might help outcomes, but more data is needed. Also, any dietary restriction strategy has to consider the patient’s overall condition: cancer patients often struggle to maintain weight, so aggressive amino acid restriction could be risky.

#### 6.5.2 BCAA Supplementation

On the flip side, adding BCAAs to the diet might help in contexts where the goal is to boost the immune system or prevent muscle wasting (cachexia). For example, in cachectic patients or those recovering from surgery, BCAA supplements can help maintain muscle mass [115,116]. In oncology, oral BCAA formulations have been given to patients with advanced liver cirrhosis (who often develop HCC)—some studies report improved survival and lower HCC recurrence, attributed to better liver function and possibly an anti-tumor milieu due to improved insulin sensitivity and reduced inflammation [117]. There’s also an intriguing idea of giving a burst of BCAAs around the time of immunotherapy to “supercharge” T cells (as suggested by the study where BCAA supplementation improved anti-PD-1 therapy in mice) [118].

The key to dietary interventions is personalization. We may one day use metabolic biomarkers to decide: does this patient’s tumor require a leucine-restricted diet, or could they benefit from extra BCAAs to support immune function? It might even be a sequential thing, e.g., restrict BCAAs to shrink a tumor, then refeed BCAAs during immunotherapy to activate T cells. These approaches will require close monitoring and are likely adjuncts to standard treatments, not stand-alones.

### 6.6 Immunometabolic Strategies and Combination Therapies

One of the most exciting emerging areas is combining BCAA-targeted approaches with other therapies, especially immunotherapies. The rationale is clear: tackle the tumor’s metabolism while simultaneously attacking it with immune or targeted agents, hitting it from multiple sides.

#### 6.6.1 Combining with Checkpoint Inhibitors

As noted, a LAT1 inhibitor might starve the tumor and also relieve some nutrient competition, possibly allowing T cells to function better. In a scenario where a tumor is only partially responsive to anti-PD-1 therapy, adding a metabolic inhibitor could tip the balance. Of course, one must ensure the immune cells are not starved in the process—so timing or dosing could be adjusted (maybe dose the metabolic drug in a pulsed manner separate from when T cells need nutrients).

#### 6.6.2 Combining with Cell Therapies

For CAR-T cells targeting solid tumors, one approach is to engineer the CAR-T’s metabolism (like the BCKDK-knockout CAR-T cells, which showed superior efficacy) [12]. These cells are essentially their own metabolic therapy—they carry an intrinsic modification that lets them function in a low-leucine environment. There’s also research on giving CAR-T patients a special diet or supplements before infusing the CAR-T cells, to get those T cells in peak metabolic shape.

#### 6.6.3 Combining with Targeted Drugs or Chemo

Another idea is that metabolic therapy might extend the efficacy of targeted drugs. For example, an EGFR inhibitor plus a BCAT1 inhibitor tackled two separate resistance mechanisms in lung cancer models and achieved deeper tumor regressions than either alone [11]. Similarly, imagine an mTOR inhibitor (to blunt growth signaling) combined with a LAT1 inhibitor (to cut off the nutrient supply)—the tumor might find it hard to escape both. Chemo might work better if given after weakening the tumor with a dietary intervention or a BCKDK inhibitor that loads the tumor cells with oxidative stress.

#### 6.6.4 Safety and Timing

Combining therapies always raises concerns of additive toxicity. Will a patient on a LAT1 inhibitor and immunotherapy experience heightened side effects, perhaps because their immune cells are more active and also nutrient-limited? Or could a low-protein diet during chemo cause patients to lose too much weight? These are real considerations, and early-phase trials will need to incorporate nutritional and metabolic monitoring as part of their design.

Yet, the potential pay-off is big. As we increasingly categorize tumors by their metabolic phenotypes (some are “BCAA-hungry”, others not so much), we can tailor a combination approach. For instance, a “BCAA-hungry, immunologically cold” tumor might get a LAT1 inhibitor + checkpoint inhibitor to simultaneously starve the tumor and invigorate immune attack [119].

### 6.7 Future Directions and Challenges

Targeting cancer metabolism, including BCAA pathways, comes with unique challenges. Unlike a mutated oncogene, which might be unique to a tumor, metabolic enzymes are also present in normal tissues. Thus, on-target side effects are a concern. BCAAs are vital for normal muscle, brain, and immune function. Indeed, patients with rare inborn errors in BCAA metabolism (like MSUD) show how essential balanced BCAA levels are—those patients require meticulous dietary management for life. The hope is that cancer cells’ addiction to BCAAs is greater and more indispensable than normal cells’ usage. So far, clinical experiences like the JPH203 trial suggest that short-term inhibition of BCAA pathways can be tolerated in adults, but long-term effects need observation [120]. One way to maximize safety and efficacy is through precision medicine approaches.

#### 6.7.1 Biomarker-Driven Selection

Before giving a BCAA metabolism drug, we should ideally have evidence that the patient’s tumor is reliant on that pathway. This could be through immunohistochemistry (e.g., LAT1 or BCAT1 staining), gene expression profiling, or metabolomic analysis of a biopsy. Patients without the metabolic target would be spared the therapy and any potential toxicity.

#### 6.7.2 Metabolic Imaging

New PET tracers are being tested, such as radiolabeled leucine or analogs that specifically get taken up by LAT1. If you scan a patient and see the tumor lights up with a LAT1 tracer, that’s a good indication that LAT1 inhibition could work. Similarly, one might monitor a patient on therapy to see if the tracer uptake goes down, indicating target engagement.

#### 6.7.3 Combination Strategies

As discussed, combining metabolic therapy with other treatments will likely be necessary for maximal effect. But combinations also offer a chance to mitigate toxicity: for instance, perhaps a lower dose of a metabolic drug can be used if it synergizes strongly with an immunotherapy, reducing the side effect risk from either alone.

#### 6.7.4 Patient Monitoring

If a patient is on a BCAA metabolism drug or diet, clinicians will need to monitor things like blood amino acid levels, liver function (since the liver handles amino acid metabolism), muscle mass (to catch any muscle atrophy early), and neurological status. Many of these can be done with routine blood tests or imaging and are not too burdensome if one knows to look for them.

Looking ahead, the field of cancer metabolism is rapidly intersecting with immunology and genomics. Multi-omics data (combining genomics, transcriptomics, and metabolomics) are helping us map out which cancers depend on which nutrients. As this map becomes clearer, therapies like the ones described in this review will find their place in the standard of care. It’s conceivable that in a future tumor board meeting, alongside discussing a patient’s tumor mutation status, the doctors will also discuss the tumor’s metabolic profile—and prescribe a drug like a LAT1 inhibitor or a special diet as part of the treatment plan.

## 7 Conclusions

Branched-chain amino acid metabolic reprogramming has emerged as a fundamental aspect of cancer biology, influencing tumor cell behavior, the tumor immune microenvironment, and responses to therapy. As discussed, cancers of the breast, lung, and many other tissues rewire BCAA uptake and catabolism to fuel their growth and adapt to environmental challenges. Molecularly, this reprogramming involves coordinated changes in transporters (like LAT1), enzymes (BCAT1/2, BCKDH, BCKDK), and signaling pathways (mTORC1 and beyond) that together promote tumor proliferation, survival under stress, and metastasis. At the same time, these alterations can disarm immune cells by depriving them of crucial nutrients or by creating an inhibitory metabolic milieu. The dual roles of BCAA metabolism—nurturing the tumor while subverting immunity—underscore its importance as a target for cancer therapy.

The study of BCAA metabolic reprogramming in cancer has evolved from biochemical curiosity to the forefront of translational research. It bridges our understanding of fundamental cellular metabolism with the clinical realities of cancer progression and therapy resistance. In this review, we have highlighted how deeply intertwined BCAA metabolic pathways are with cancer’s molecular machinery and the immune contexture. The evidence to date strongly suggests that therapeutically targeting BCAA metabolism—in a cancer-specific, precision manner—can open new avenues for combating malignancies, particularly those that currently evade effective treatments. As research continues to unravel remaining mysteries (such as the long-term consequences of BCAA modulation and the best ways to integrate these strategies into standard care), we remain optimistic that leveraging this metabolic vulnerability link will improve outcomes of patients across multiple cancer types.

## Data Availability

Not applicable.

## Abbreviations

The following abbreviations are used in this manuscript
AML: Acute myeloid leukemia
ATP: Adenosine triphosphate
α-KG: α-ketoglutarate
BCAAs: Branched-chain amino acids
BCATs: Branched-chain amino acid transferase
BCKAs: Branched-chain α-ketoacids
BCKDH: Branched-chain α-ketoacid dehydrogenase complex
CAR-T: Chimeric antigen receptor T-cell immunotherapy
EB: Eupalinolide B
EGFR: Epidermal growth factor receptor
ER: Estrogen receptor
GSH: Glutathione
HCC: Hepatocellular carcinoma
HER2: Human epidermal growth factor receptor 2
HRR: Homologous recombination repair
IDH: Isocitrate dehydrogenase
IDO: Indoleamine 2, 3-dioxygenase
IFNγ: Interferon gamma
IL-15: Interleukin 15
LAT1: L-type amino acid transporter 1
MDSCs: Myeloid-derived suppressor cells
mTORC1: Mammalian target of rapamycin complex 1
NADPH: Nicotinamide adenine dinucleotide phosphate
NF-κB: Nuclear factor kappa-B
NK: Natural killer
NSCLC: Non-small cell lung cancer
PDAC: Pancreatic ductal adenocarcinoma
PD-1: Programmed cell death protein 1
PR: Progesterone receptor
RNF8: Ring finger protein 8
ROS: Reactive oxygen species
TAMs: Tumor-associated macrophages
TCA cycle: Tricarboxylic acid cycle
TKIs: Tyrosine kinase inhibitors
TME: Tumor microenvironment
TNBC: Triple-negative breast cancer
